# Subjective and Objective Evaluation of a Novel Nasal Tip Elevating Strip for Nasal Obstruction: A Pilot Study

**DOI:** 10.7759/cureus.113709

**Published:** 2026-07-31

**Authors:** Matteo Pezzoli, Andrea Manca, Umberto Visentin, Marzia Ariano, Alessandro Pagliassotto

**Affiliations:** 1 Ear, Nose, and Throat, Martini Hospital, Turin, ITA

**Keywords:** airway resistance, nasal obstruction, nasal septum, nasal valve, patient reported outcome measures

## Abstract

Background

Chronic nasal obstruction represents a significant clinical challenge in rhinology, frequently leading to a reduction in respiratory function and a measurable impact on patient-reported quality of life. While external nasal dilators are frequently utilised as a non-invasive treatment, most existing devices focus solely on lateral nasal valve stabilisation. A novel biomechanical approach involving nasal tip elevation may offer additional symptomatic relief, particularly as a temporary measure for patients awaiting definitive surgical correction. The objective of this pilot study was to evaluate the clinical impact of a novel biomechanical approach, based on nasal tip elevation, on both patient-reported symptoms and objective inspiratory airflow.

Materials and methods

A prospective study was conducted between December 2025 and June 2026 on 29 Caucasian adults (mean age 42.86 ± 11.09 years) with severe nasal septal deviation and inferior turbinate hypertrophy. Objective patency was assessed using peak nasal inspiratory flow (PNIF). Subjective outcomes were evaluated via a visual analog scale (VAS) for breathing ease at day one, and nasal obstruction symptom evaluation (NOSE) and Epworth sleepiness scale (ESS) questionnaires after seven days of treatment.

Results

NOSE scores diminished significantly after one week (from 12.41 ± 3.88 to 10.45 ± 3.52; p < 0.05). VAS scores demonstrated an improvement trend that did not reach statistical significance (p = 0.07). No significant changes were registered in PNIF or ESS values between baseline and the end of the treatment period. No major complications occurred; only one patient (3.4%) noted minor skin redness at the application site, with no study dropouts.

Conclusions

The novel nasal tip-elevating strip provides significant symptomatic relief in patients with chronic nasal obstruction. It represents an effective non-invasive tool for symptomatic management and a reliable bridge therapy for individuals awaiting definitive surgical correction.

## Introduction

Nasal strips can be used as a non-invasive treatment for nasal obstruction [1]. This is a common condition that affects nearly 30% of the population, often significantly diminishing quality of life, physical performance, and sleep quality [2,3]. In daily clinical practice, Ear, Nose, and Throat (ENT) specialists are frequently consulted by patients who ask whether over-the-counter nasal strips are truly effective or merely a temporary placebo. The mechanical challenge of nasal wall collapse is well-documented in medical literature; instruments designed to prevent it were described as early as the beginning of the 1900s by Francis [4].

However, external nasal strips only gained widespread popularity in the 1990s following clinical validation of their effect on the nasal valve area [5]. Despite their popularity, the scientific consensus remains divided. While some research has suggested a positive effect on recovery and performance for athletes, other studies found no statistical difference in ventilation, heart rate, or lactic acid concentration during exercise [6].

As recently reviewed by Ahmed et al. [2], the current landscape of non-surgical options includes external devices such as the standard Breathe Right strips (Foundation Consumer Healthcare, Pittsburgh, USA), the Asymmetric Butterfly prototype, and the Master-Aid Roll-Flex (Pietrasanta Pharma S.p.A., Capannori, Italy), as well as internal solutions. Most of these tools focus primarily on the nasal walls. Unlike conventional strips that focus solely on the valve area [5], the device we have chosen to analyze is specifically designed to mechanically elevate the nasal tip. A similar concept was evaluated by Ottaviano et al. [7], but that study utilized a custom-made strip. The goal of this study was to assess the clinical utility of this specific tip-elevating mechanism as a non-invasive symptomatic treatment for nasal obstruction in a selected cohort of surgical candidates.

## Materials and methods

Study design, ethics, and timeframe

This was a prospective, single-centre, interventional pilot study designed to evaluate the efficacy of a novel nasal tip-elevating dilator strip. The research was conducted at the ENT Department of Martini Hospital, ASL City of Turin, Italy, between December 2025 and June 2026. The clinical protocol was designed in accordance with the Declaration of Helsinki and received formal approval from the Inter-Institutional Ethics Committee (Comitato Etico Interaziendale; Protocol Number CET00222/2025). Before enrolment, all participants provided written informed consent after a detailed explanation of the study’s purpose, procedures, and potential risks.

Participant selection and criteria

A total of 29 Caucasian adult patients (21 males and eight females) awaiting functional nasal surgery for septoplasty and inferior turbinate reduction were consecutively recruited. The mean age of the overall sample was 42.86 ± 11.09 years, with a range of 22 to 62 years, as detailed in Table 1. The inclusion criteria comprised patients with chronic nasal obstruction secondary to nasal septal deviation and inferior turbinate hypertrophy who had failed maximal medical therapy. Failure of medical therapy was defined as the continuous use of intranasal corticosteroids, specifically Mometasone furoate (50 μg, two sprays per nostril once a day) or Budesonide (100 μg, two sprays per nostril once a day), for at least one month before study entry. Furthermore, participants were required to demonstrate the ability to correctly perform the Peak Nasal Inspiratory Flow (PNIF) manoeuvre and possess an adequate understanding of patient-reported outcome measures. Participants were excluded if they had a history of alcohol or substance abuse within the past two years, a known allergy to nasal strip materials or adhesives, or active facial skin conditions such as eczema or dermatitis. Finally, patients with clinical instability, including uncontrolled asthma, acute respiratory infections, or recent nasal trauma, were also excluded. Additional exclusion criteria included nasal polyposis, symptomatic concha bullosa, or other endonasal space-occupying lesions detected during the initial endoscopic screening.

**Table 1:** Demographic characteristics and clinical outcomes of the study population (n = 29). The table provides a comprehensive summary of participant data and a comparison between baseline values and results obtained after the application of the nasal tip-elevating strip at baseline. The results of the questionnaire at baseline and after 7 days (T1). Data are presented as mean ± standard deviation or as absolute frequencies and percentages. VAS: visual analog scale; PNIF: peak nasal inspiratory flow; ESS: Epworth sleepiness scale; NOSE: nasal obstruction symptom evaluation.

Clinical Parameter	Without Strip	With Strip	After 7 Days (T1)	t-value (Test Statistic)	p-value
VAS (mean, SD)	5.41±2.05	4.59 ±2.28	—	1.88	0.07
PNIF (mean,SD)	86.96 ±57.50	98.97 ±60.31	—	1.31	0.20
ESS score (mean, SD)	8.93 ±5.03	—	8.48 ±4.99	0.91	0.37
NOSE score (mean, SD)	12.41 ±3.88	—	10.45 ±3.52	2.47	0.02

The sample size of 29 participants was determined based on the exploratory nature of this pilot study. Given the device's novel biomechanical approach, this cohort size was deemed scientifically sufficient to detect clinically meaningful changes in subjective obstruction scores (NOSE scale) and to provide preliminary data for future large-scale trials. This approach aligns with previous pilot investigations evaluating external nasal dilators in similar surgical candidate populations [7].

The novel device and interventional protocol

**Figure 1:**
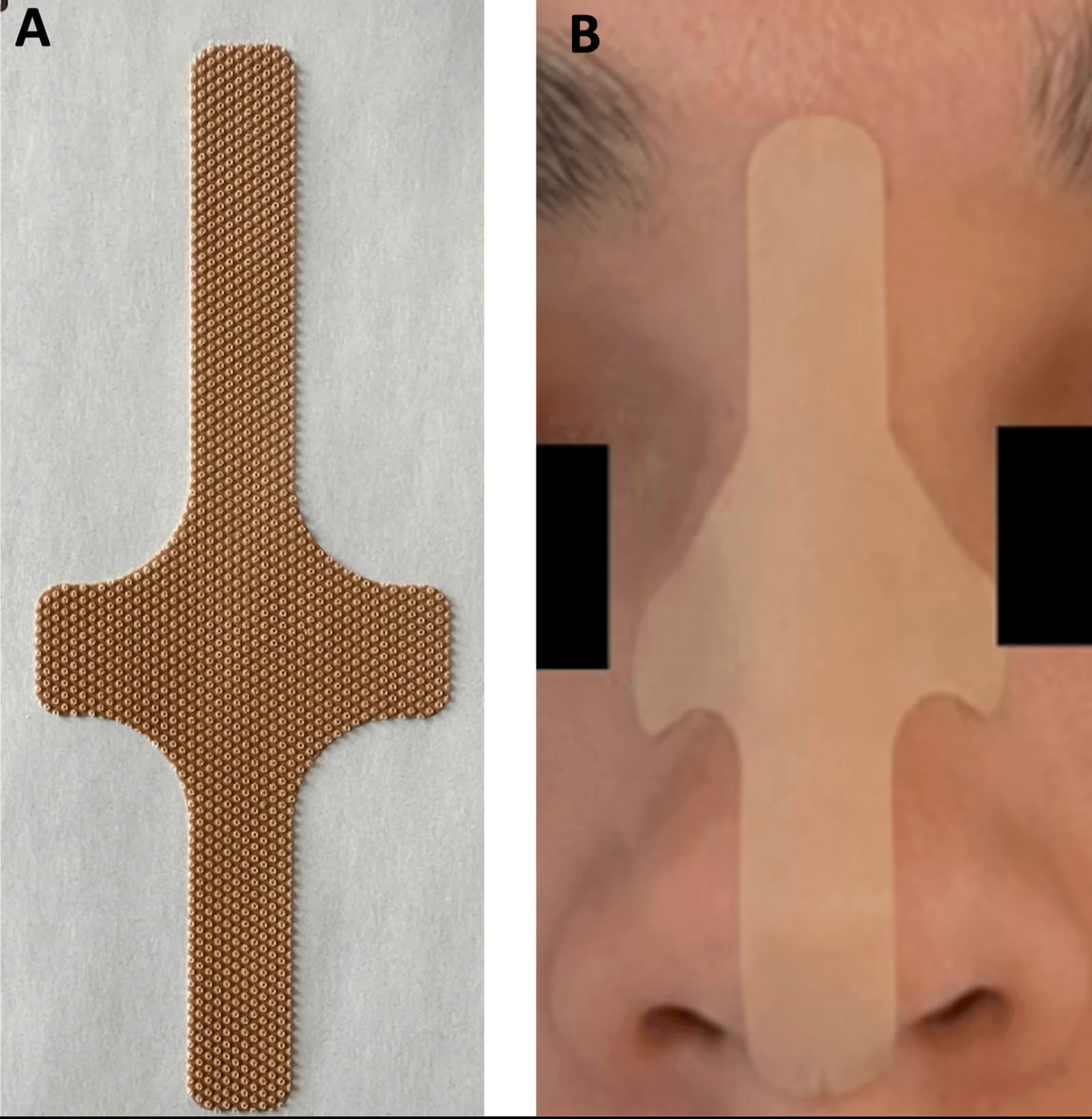
A. Nasal tip elevating device; B. It's application.

The medical device under investigation was a non-invasive, CE-marked nasal dilator strip (CR8 RESPIRA, Euprana SRL, Turin, Italy), which is illustrated in Figure 1. Participants were instructed to utilise the strip for seven consecutive days under two specific conditions: at night during sleep and during bouts of physical exercise. The strip was applied according to the manufacturer’s instructions on the anterior portion of the nasal dorsum to achieve a mechanical lift of the nasal tip while ensuring lateral wall stabilisation. To maintain local hygiene and ensure optimal adhesive force and mechanical efficacy, each participant was provided with a supply of single-use disposable strips; instructions were given to apply a fresh device before each nocturnal sleep period and each physical exercise session throughout the seven-day interventional period. Tolerability and safety were monitored via adverse event reporting during the follow-up visit on day seven.

Objective assessment of nasal patency

Objective nasal patency was assessed using a standardised PNIF metre (In-check, Clement Clarke International, UK), which is a validated manoeuvre for detecting nasal valve collapse [8,9]. Measurements were performed at baseline and on day one after the application of the strip. Following established protocols, PNIF was measured with an adequate mask seal while instructing patients to inhale sharply and forcibly through the nose, with the mouth firmly closed, for at least one second. The highest of three consecutive measurements was recorded for analysis. To ensure diagnostic accuracy, participants manoeuvred for both seated and supine positions.

Subjective outcome measures

Subjective efficacy was quantified using a battery of validated psychometric instruments. At the time of the initial visit, a visual analog scale (VAS), which has been previously validated for the assessment of clinical symptoms [7], was utilised for a real-time subjective evaluation of breathing ease (0-100 mm scale: 0 = "extremely open", 100 = "completely blocked"). Patients were also required to complete baseline and day 7 questionnaires, including the Nasal Obstruction Symptom Evaluation (NOSE) scale, a 5-item disease-specific tool [3], and the Epworth Sleepiness Scale (ESS), an 8-item scale used to assess daytime sleepiness [10].

Statistical analysis

Data normality was assessed using the Shapiro-Wilk test. Differences between baseline and the use of strips for VAS and PNIF, as well as pre- vs. post-treatment differences for NOSE and ESS scores, were calculated using the paired sample t-test. All statistical analyses were performed using GraphPad Prism software, version 9.0 (GraphPad Software, San Diego, CA, USA). Statistical significance was set at p < 0.05.

## Results

Participant demographics and general outcomes

A total of 29 Caucasian adult patients, consisting of 21 males and eight females, met the inclusion criteria and completed the study protocol. The mean age of the overall sample was 42.86 ± 11.09 years, with a range of 22 to 62 years. A comprehensive summary of the participant demographics and the clinical data recorded during the study is provided in Table 1.

Clinical outcomes and subjective assessments

**Figure 2:**
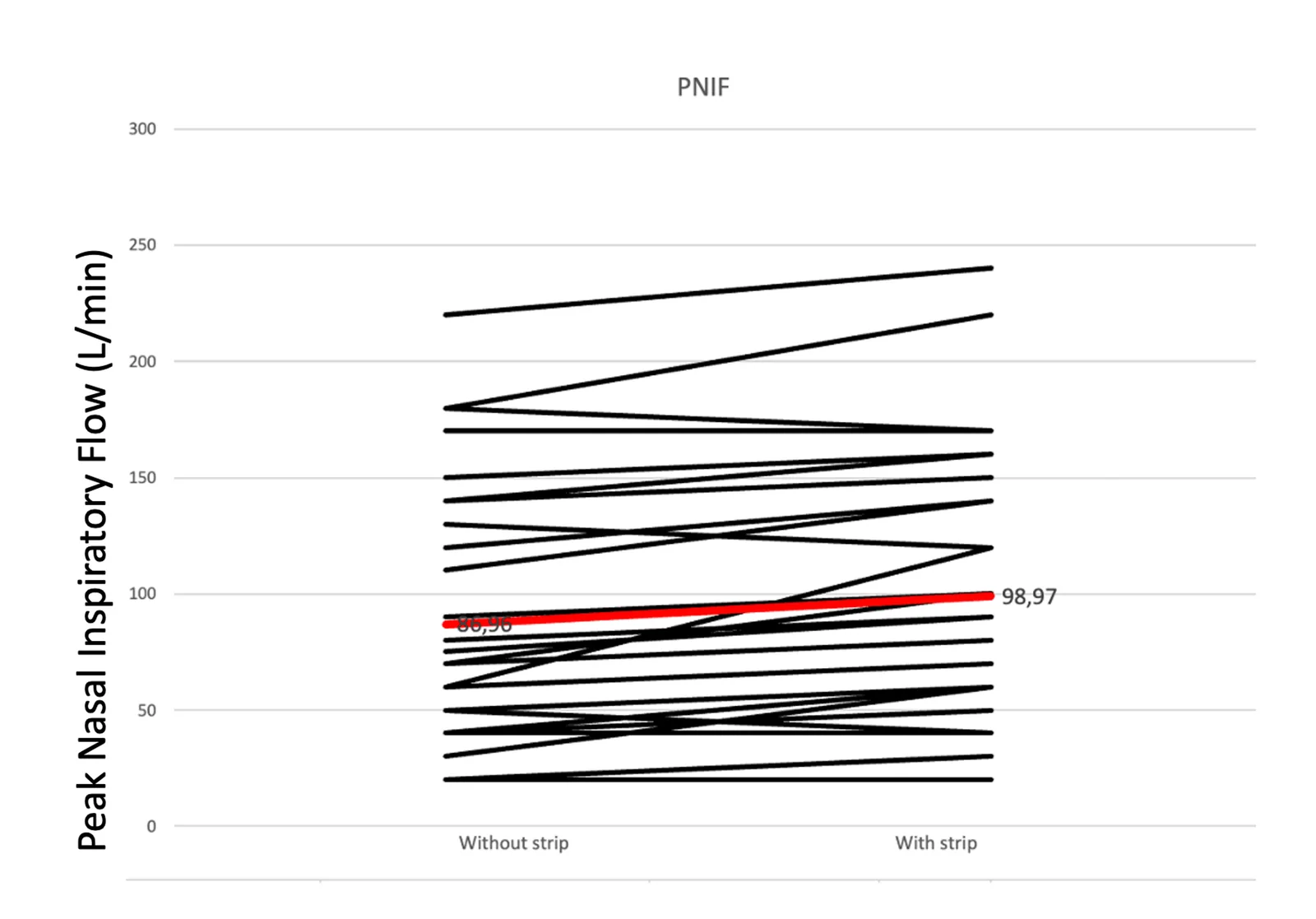
Individual changes in Peak Nasal Inspiratory Flow (PNIF) values between baseline (Without strip) and after application (With strip). The red line represents the mean variation of the cohort.

**Figure 3:**
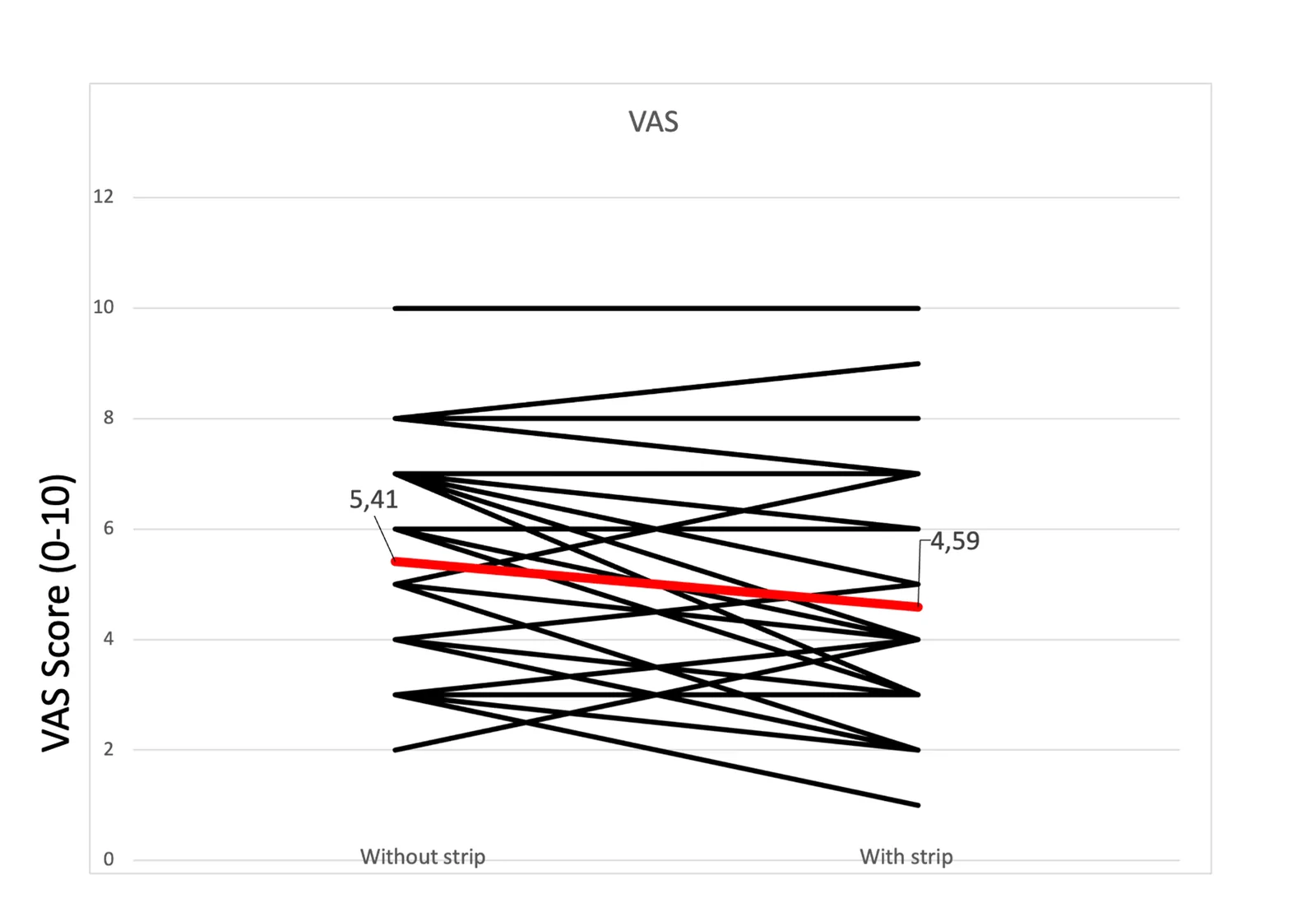
Individual changes VAS Score values between baseline (Without strip) and after application (With strip). The red line represents the mean variation of the cohort.

**Figure 4:**
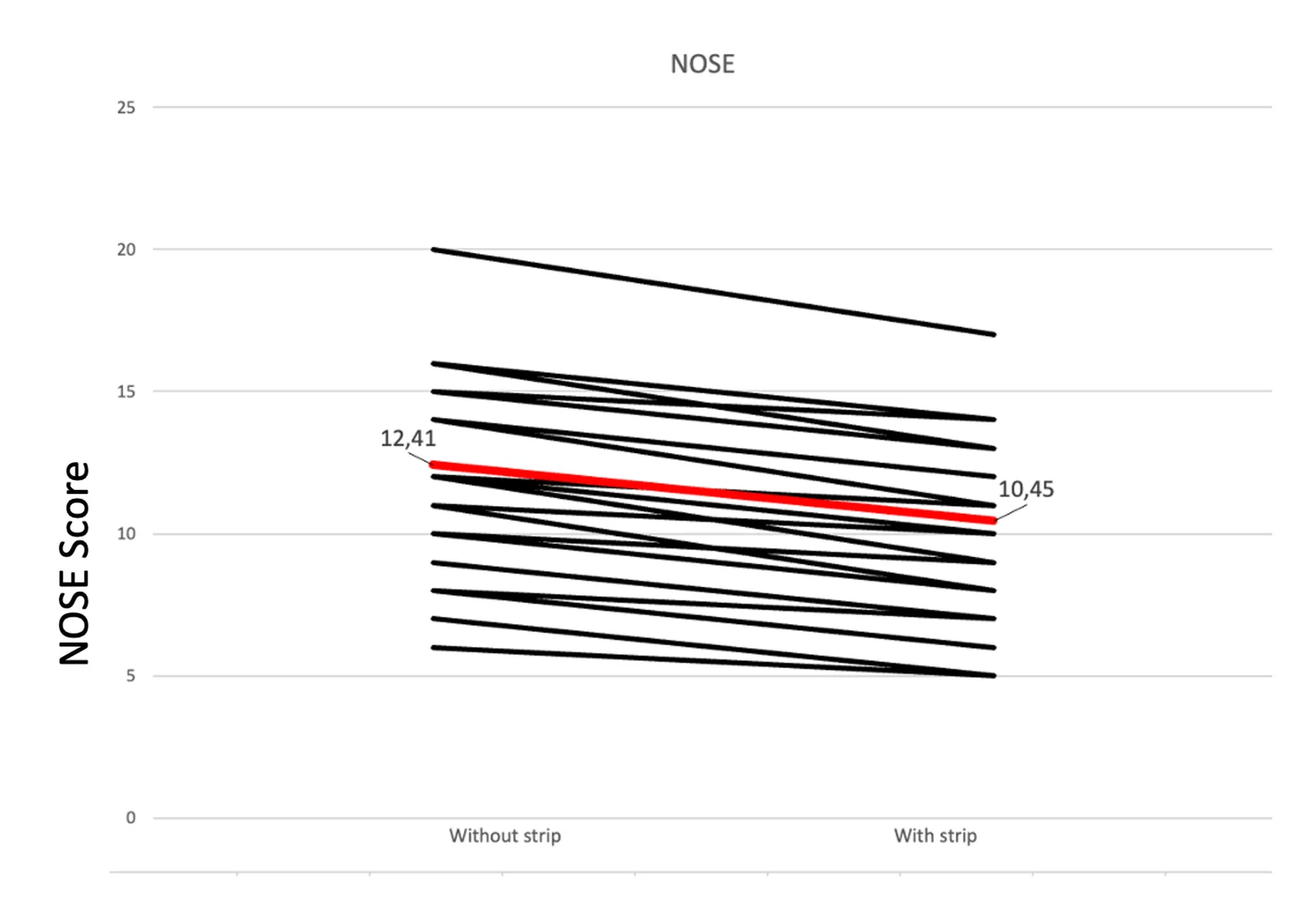
Variation in Nasal Obstruction Symptom Evaluation (NOSE) scores from baseline to Day 7. A statistically significant reduction in subjective obstruction was registered (p < 0.05).

**Figure 5:**
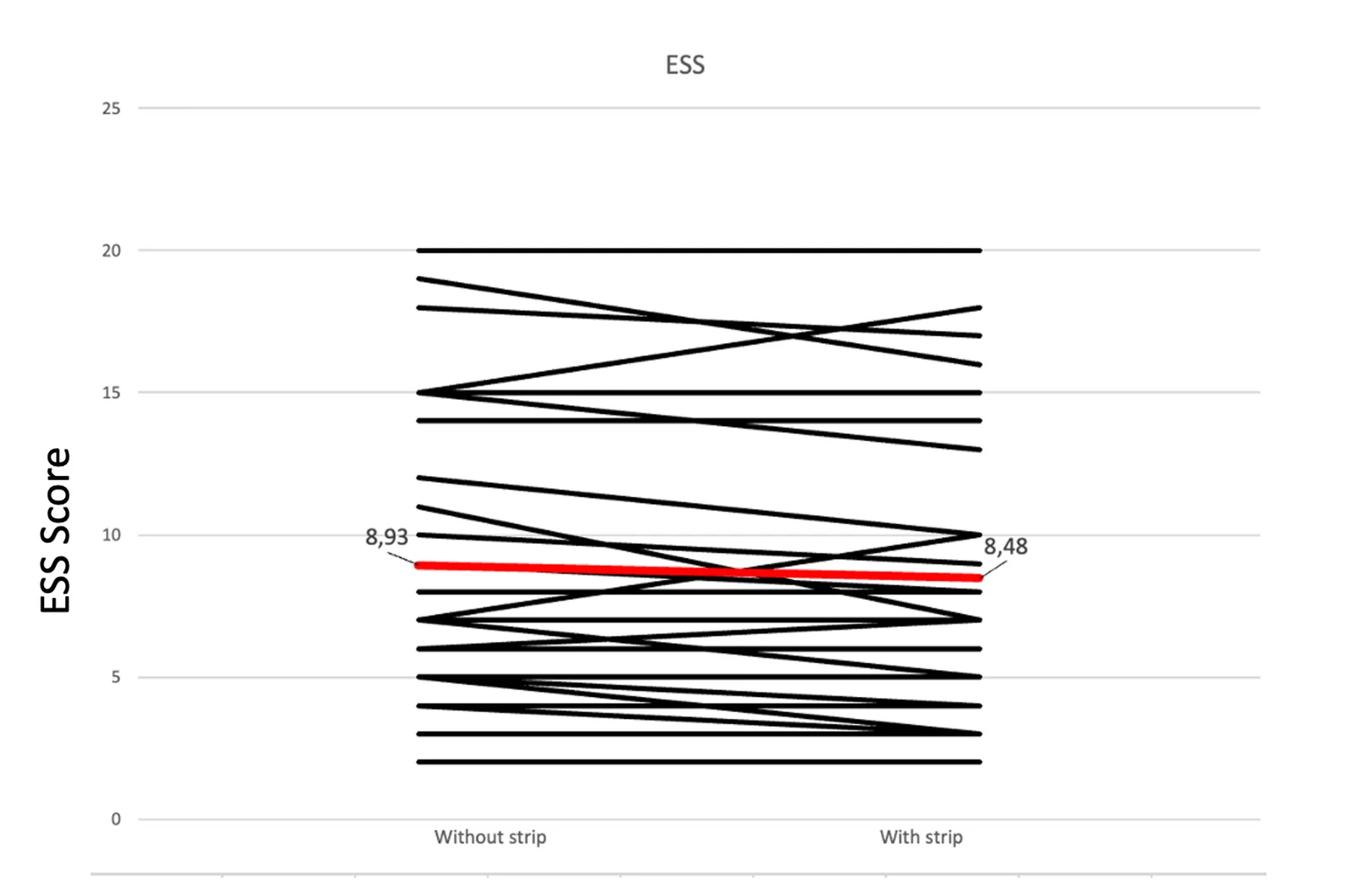
Comparison of Epworth Sleepiness Scale (ESS) scores before and after 7 days of treatment, showing no significant impact on daytime sleepiness.

Regarding the immediate objective measurements registered on day one, the data demonstrated no statistically significant relationship between the application of the novel nasal tip-elevating strip and an improvement in PNIF, as illustrated in Figure 2. Similarly, the VAS scores for breathing ease showed a trend toward improvement that did not reach statistical significance (p = 0.07), with individual patient variations shown in Figure 3. In contrast, the clinical outcomes after seven days of continuous treatment revealed that the subjective perception of nasal obstruction, assessed via the NOSE scale, diminished significantly (p < 0.05). The reduction in NOSE scores is further detailed in Figure 4. However, the ESS scores did not show any significant variation or improvement during the one-week treatment period, as depicted in Figure 5.

Safety and tolerability

The device was generally well-tolerated, and no major complications, such as severe allergic reactions or skin lacerations, were registered during the clinical evaluation. Minor application site reactions were infrequent; only one patient (3.4%) noted slight skin redness and discomfort. This adverse event was classified as minor and did not lead to treatment dropout or discontinuation of the interventional protocol.

## Discussion

Principal findings 

The results of the present study demonstrate that the application of a novel nasal tip-elevating strip leads to a significant improvement in the subjective perception of nasal obstruction, as evidenced by the reduction in NOSE scores. In contrast, no statistically significant changes were registered in objective parameters, such as PNIF, or in daytime sleepiness, as measured by the ESS. In accordance with the literature, external nasal dilator strips have been extensively investigated for the treatment of nasal valve collapse and snoring. However, most previous studies focused on conventional devices designed solely for lateral wall stabilisation, such as the Breathe Right strips [5,11]. To our knowledge, the only other report evaluating a mechanism specifically aimed at nasal tip elevation utilised a custom-made strip manually cut from a commercial adhesive roll [7]. Therefore, the device analysed in this study represents a novel tool in the non-surgical management of nasal obstruction.

Objective assessment and the 'stenting effect'

Regarding objective assessment, we utilised PNIF, which has been reported as a reliable tool with a power to discriminate nasal obstruction similar to that of traditional rhinomanometry [12]. It is worth underlining that PNIF offers a distinct clinical advantage: it does not require the use of nasal adapters or plugs that are necessary in rhinomanometry and acoustic rhinometry. These adapters can often distort the nasal valve anatomy, creating a so-called stenting effect that artificially stabilises the valve and distorts the area of maximum resistance [13]. In contrast, the PNIF utilised in our protocol employs a face mask that avoids any direct contact with the nasal vestibule, thus preventing anatomical distortion and allowing for a more reliable evaluation of dynamic valve collapse [8]. However, it must be noted that PNIF remains an effort-dependent measurement and does not provide information on nasal resistance throughout the entire respiratory cycle [14].

Comparison with existing literature and cohort characteristics

The PNIF values registered in our cohort were generally lower than those reported in other studies [7,15]. This difference can be explained by the fact that, unlike the subjects in previous reports who were often healthy volunteers, our patients were all affected by severe nasal septal deviation and were already scheduled for surgical intervention. This highlights the importance of considering the severity of anatomical obstruction when evaluating mechanical dilators. Regarding the subjective results, the NOSE score diminished significantly in our sample. This clinical improvement is in accordance with several studies demonstrating that even when objective parameters remain unchanged, patients perceive a meaningful reduction in obstruction symptoms with mechanical dilators [2,7].

Clinical implications for daytime sleepiness and obstructive sleep apnea syndrome (OSAS)

Conversely, the ESS scores did not show significant variation. It is reasonable to suggest that nasal strips have a limited role in managing daytime sleepiness, as nasal obstruction is only one component of sleep-disordered breathing. Indeed, a meta-analysis by Camacho et al. [16] and recent findings by Alotaibi et al. [17] concluded that nasal dilators do not meaningfully improve outcomes in patients with OSAS, as they do not address the dynamic pharyngeal collapse that characterises the disease. The clinical effectiveness of this intervention is underscored by the significant improvement in subjective obstruction scores, despite the lack of measurable changes in static peak flow or daytime sleepiness. In patients with pronounced structural obstruction awaiting surgery, mechanical dilators may primarily enhance dynamic nasal valve stability during inspiration, thus providing meaningful symptomatic relief without necessarily altering static resistance. It is important to acknowledge the discrepancy between the significant subjective improvement observed via the NOSE scale and the lack of statistically significant changes in objective PNIF measurements. This suggests that the patient's perception of relief may be driven by subtle dynamic changes in the nasal valve area that are not fully captured by static peak flow metrics. For a non-invasive 'bridge therapy', the patient’s perceived ease of breathing represents a primary and clinically relevant endpoint that justifies its use in the pre-operative period

Limitations of the study

Finally, this study has several limitations that warrant consideration. The primary constraint involves the relatively small sample size and the highly selected nature of our cohort, as participants were limited to patients with severe nasal obstruction refractory to medical therapy. While this specific profile allowed us to evaluate the device under conditions of significant anatomical resistance, it may limit the generalizability of the findings to the broader population. Furthermore, the absence of polysomnographic data precludes definitive conclusions regarding the device’s impact on objective snoring parameters or pharyngeal dynamics. Although subjective relief was reported, an objective correlate for sleep-disordered breathing remains unexplored in this cohort.

Furthermore, the open-label, single-arm nature of this study, combined with the lack of a control or placebo group, represents a potential source of bias that limits the generalizability of our findings

## Conclusions

The results of this study suggest that the novel nasal tip-elevating strip may represent a promising, significant symptomatic relief for patients suffering from chronic nasal obstruction. The measurable reduction in NOSE scores demonstrates the clinical efficacy of this device as a non-invasive tool for managing obstructive symptoms in a real-world clinical setting. Based on these findings, the strip represents a reliable bridge therapy for symptomatic management in individuals awaiting definitive functional nasal surgery, such as septoplasty and inferior turbinate reduction. In conclusion, although the device did not significantly alter objective airflow parameters (PNIF) or daytime sleepiness (ESS) in this severely obstructed cohort, it remains a valuable conservative option for the immediate relief of subjective nasal blockage. Further research in broader populations is warranted to establish the long-term utility of this mechanical approach.
